# Application of principal component analysis and logistic regression model in lupus nephritis patients with clinical hypothyroidism

**DOI:** 10.1186/s12874-020-00989-x

**Published:** 2020-05-01

**Authors:** Ting Huang, Jiarong Li, Weiru Zhang

**Affiliations:** Department of Rheumatology, Xiangya Hospital, Central South University, Xiangya Road, Changsha, 410000 Hunan China

**Keywords:** Logistic regression, PCA, Lupus nephritis, Hypothyroidism

## Abstract

**Background:**

Previous studies indicate that the prevalence of hypothyroidism is much higher in patients with lupus nephritis (LN) than in the general population, and is associated with LN’s activity. Principal component analysis (PCA) and logistic regression can help determine relevant risk factors and identify LN patients at high risk of hypothyroidism; as such, these tools may prove useful in managing this disease.

**Methods:**

We carried out a cross-sectional study of 143 LN patients diagnosed by renal biopsy, all of whom had been admitted to Xiangya Hospital of Central South University in Changsha, China, between June 2012 and December 2016. The PCA–logistic regression model was used to determine the influential principal components for LN patients who have hypothyroidism.

**Results:**

Our PCA–logistic regression analysis results demonstrated that serum creatinine, blood urea nitrogen, blood uric acid, total protein, albumin, and anti-ribonucleoprotein antibody were important clinical variables for LN patients with hypothyroidism. The area under the curve of this model was 0.855.

**Conclusion:**

The PCA–logistic regression model performed well in identifying important risk factors for certain clinical outcomes, and promoting clinical research on other diseases will be beneficial. Using this model, clinicians can identify at-risk subjects and either implement preventative strategies or manage current treatments.

## Background

Systemic lupus erythematosus (SLE) is a multisystem autoimmune disease, and lupus nephritis (LN) is a frequently occurring and serious complication of SLE [1, 2]. Studies indicate that the prevalence of hypothyroidism is much higher in SLE, and especially among LN patients, than in the general population [3–6]; additionally, the risk of subsequent cardiovascular events and renal impairment is higher among LN patients with thyroid dysfunction. Accordingly, analysis of the associations between LN and hypothyroidism and a determination of relevant risk factors would greatly aid in diagnosis and disease management.

However, the pathological and physiological mechanisms underlying SLE with hypothyroidism are sophisticated. Furthermore, the availability of multiple indicators and of large relevant datasets makes it difficult to analyse clinical data directly; therefore, the precise nature of these mechanisms remains unknown [6–8].

Logistic regression is widely used to analyse the relationship between individual risk/protective factors and outcomes [9]. However, if the variables therein are collinear, the regression equation will be unstable and its results difficult to predict. Principal component analysis (PCA) is a powerful method by which to explore intricate datasets that feature multiple variables. PCA uses a mathematical algorithm to determine a smaller number of new variables called principal components (PCs), which are linear functions of those in the original dataset. Hence, PCA scales down the dimensionality of a large dataset while preserving as much statistical information as possible [10, 11]. As such, the current study’s use of PCA helps ensure the stability of the regression equation. In fact, PCA has previously been used to analyze complex serological and immunological datasets with multiple variables in SLE cross-sectional studies. Raymond et al [12] used PCA to describe the dynamic interplay and the influence of complex cytokines measured in serum, detect the cytokine groups that differentiated across disease activity in SLE patients. Adel Helmy et al. [13] used PCA to identify cytokine groups which accounted for the majority of the variation within the serological laboratory test data in traumatic brain injury patients.

The current study examines the laboratory test results of selected patient populations, and leverages PCA–logistic regression analysis to pinpoint key PCs. Such information may greatly assist in the prevention or management of this disease.

## Methods

### Patients

In our cross-sectional study, we investigated 143 LN patients diagnosed through renal biopsy who had been admitted to Xiangya Hospital of Central South University in Changsha, China during the June 2012–December 2016 period. The exclusion criteria included the coexistence of another autoimmune disease or having been diagnosed with thyroid disease prior to LN. All patients were informed of the objectives of this study, and each provided signed written consent prior to enrolment. As this research did not affect patient treatment, as per Central South University policies, ethics board approval was not required.

### Collection of clinical data

Data on patient characteristics, clinical symptoms, and laboratory results were retrospectively collected from each patient’s medical records. These included: (1) general information, including age and sex; (2) clinical symptoms, including course of disease, hypertension, fever, cutaneous manifestations, alopecia, oral ulcer, malar rash, renal dysfunction (proteinuria), and haematological disease; and (3) laboratory results, including white blood cell count, haemoglobin (Hb) concentration, concentration of total protein (TP), serum lipid, erythrocyte sedimentation rate, C-reactive protein, C3, C4, and antibodies to dsDNA, simth, SSA, SSB, anti-U1 ribonucleoprotein, and ribosomal P protein. Patients’ SLE disease activity (i.e., SLEDAI) scores were collected from medical records and calculated by an experienced clinician.

### Statistical analysis

Values herein are expressed as mean (standard deviation), median, and interquartile range, or as a number and percentage. We undertook comparisons between categorical variables by using the *χ*^2^ test, and between continuous variables in two independent groups by using the t-test. In cases where we were unable to establish a normal distribution for a variable, we performed the Mann–Whitney U-test.

We performed PCA by using SPSS software (a factor analysis package), to determine the interplay of clinical variables among LN patients with and without hypothyroidism. We achieved convergence during an Oblimin rotation with Kaiser normalization. In the final PCA iteration, we covered nine clinical variables in the patient group analysed. To be considered a PC, a variable’s eigenvalue had to exceed 1, and PC_1_ represents the group of variables that induced the greatest amount of variation in the data. We used logistic regression to further screen clinically significant eigenvalues and scrutinize critical factors that affect outcomes among LN patients.

We performed the analysis in three stages. First, we performed a monofactor analysis to examine differences between LN patients with and without hypothyroidism. Second, we performed PCA with regard to all the serology, immunology, and biochemistry variables of LN patients. We truncated those data by rotational reorientation to maximize variance along the new axis (i.e., PC) while concurrently preserving the relationship and order among the data points; the PCs could then be used in further classification, as they retain information from the original data. Third, the absolute majority of cumulative contribution (> 2/3) was used to extract PCs as independent variables, and the clinical outcome was used as a dependent variable for logistic regression modelling. In this way, we were able to obtain the PCs that significantly correlated with certain clinical outcomes. We generated an ROC of multivariate observations to assess the PCA—logistic regression model’s performance. Statistical analysis was performed using SPSS (version 19), and all *p*-values less than 0.05 were considered statistically significant.

## Results

### Patient characteristics

We compared the clinical characteristics of 48 LN patients with hypothyroidism and 94 LN patients with euthyroidism (Table 1). The two groups were well matched in terms of age (35.6 vs. 33.1 years; *p* > 0.05), sex (87.5% vs. 83.2% female; *p* > 0.05), and disease duration (36 vs. 15 months; *p* > 0.05). LN patients with hypothyroidism had a significantly higher frequency of rash, and higher levels of serum creatinine (SCr), blood urea nitrogen (BUN), blood uric acid (UA), triglyceride (TG), and low-density lipoprotein (LDL) concentrations. Additionally, Table 1 clearly shows that the LN patients with hypothyroidism had lower Hb, C3, and C4 levels. Notwithstanding these characteristics, any analysis leveraging only a single variable would not be as accurate as comprehensive research involving multiple variables to evaluate the risk factors for LN with hypothyroidism, and the accurate selection of variables of value remains difficult. The PCA–logistic regression model we use in the current study stands as a reasonable solution to this problem.

**Table 1:** Main demographic, clinical and biochemical data of LN patients with hypothyroidism and euthyroidism

	Euthyroid(*n* = 95)	Hypothyroidism(*n* = 48)	*P*-value
Female, n(%)	79 (83.2)	42 (87.5)	0.497
Mean age (years)	33.1 ± 12.9	35.6 ± 12.0	0.660
SBP (mmHg)	128.9 ± 22.7	136.1 ± 22.5	0.568
DBP (mmHg)	83.3 ± 16.8	86.9 ± 14.8	0.899
Disease duration (months)	36 (6–108)	15 (3–69)	0.212
Fever, n (%)	9 (9.5)	2 (4.2)	0.335
**Rash, n (%)**	**39 (41.1)**	**47 (97.9)**	**0.016**
Photosensitivity, n (%)	17 (17.9)	23 (47.9)	0.617
Raynaud’s phenome, n (%)	11 (11.6)	5 (10.4)	0.835
Alopecia, n (%)	25 (26.3)	20 (41.7)	0.062
Oral ulcers, n (%)	11 (11.6)	3 (6.3)	0.475
Arthritis, n (%)	34 (35.8)	22 (45.8)	0.245
SLEDAI	10.5 ± 4.8	12.3 ± 4.4	0.330
WBC(10^9^/L)	6.3 (4.4–8.7)	5.1 (3.6–8.3)	0.124
**Hb (g/L)**	**107 (91–126)**	**95 (75–105)**	**0.001**
PLT(109/L)	198.3 ± 94.4	144.5 ± 74.5	0.096
TP (g/L)	58.1 ± 10.5	50.2 ± 11.8	0.381
ALB (g/L)	29.5 ± 7.0	21.9 ± 6.8	0.957
GLB (g/L)	28.6 ± 6.9	28.3 ± 8.8	0.070
**Scr (umol/L)**	**72 (62–107)**	**116 (81.6–208.9)**	**0.000**
**BUN (mmol/L)**	**5.2 (3.9–8.2)**	**10.7 (5.9–15.4)**	**0.000**
**UA (umol/L)**	**362.2 (294.6–463.1)**	**436.6 (342.7–568.2)**	**0.006**
**TG (mg/dl)**	**1.8 (1.3–3.0)**	**2.6 (1.8–4.0)**	**0.001**
TC (mmol/L)	5.2 (4.2–6.4)	5.7 (4.7–7.7)	0.099
HDL (mmol/L)	1.3 (0.9–1.7)	1.2 (1.0–1.5)	0.215
**LDL (mmol/L)**	**3.2 (2.4–4.0)**	**4.1 (3.0–5.1)**	**0.016**
HDL/TC	0.3 ± 0.1	0.2 ± 0.1	0.299
ESR (mm/h)	36 (26–55)	34.5 (21.5–58.5)	0.457
CRP (mg/L)	4.8 (2.3–9.8)	4.9 (1.9–10.3)	0.661
**C3(mg/L)**	**460 (361–674)**	**327.5 (328.5–440)**	**0.000**
**C4(mg/L)**	**109 (59.5–153)**	**62.1 (42.1–126.8)**	**0.049**
IgG (g/L)	12.6 (8.7–19.0)	12.4 (7.4–20.1)	0.942
IgA (g/L)	2.4 (1.5–3.2)	2.5 (1.7–3.4)	0.489
IgM (g/L)	1.0 (1.0–1.5)	0.8 (0.5–1.4)	0.248
Anti-dsDNA antibodies, n (%)	57 (60.0)	35 (72.9)	0.185
Anti-Sm antibodies, n (%)	27 (28.4)	13 (27.1)	0.715
Anti-SSA antibodies, n (%)	52 (54.7)	34 (70.8)	0.133
Anti-SSB antibodies, n (%)	13 (13.7)	6 (0.1)	0.292
Anti-U1RNP antibodies, n (%)	44 (46.3)	24 (50)	0.902
Anti-RNP antibodies, n (%)	34 (35.8)	21 (43.8)	0.260

Datas are given as mean (SD), median, and interquartile range or as number and percentage. Significant differences between two groups are indicated in bold. *SBP* systolic blood pressure, DBP diastolic blood pressure, WBC white blood cell, *Hb* haemoglobin, *PLT* platelet count, TP total protein, ALB albumin, GLB globulin, SCr serum creatinine, BUN blood urea nitrogen, UA blood uric acid, TG triglyceride, TC cholesterol, *LDL* low-density lipoprotein, *HDL* high-density lipoprotein, *ESR* erythrocyte sedimentation rate, *CRP* C-reactive protein, *Anti-dsDNA antibodies* anti-double-stranded DNA antibodies, *Anti-Sm antibodies* Anti-Smith antibodies, *Anti-U1RNP antibodies* anti-U1 ribonucleoprotein antibodies, *Anti-RNP antibodies* Anti-ribosomal P protein antibodies, *SLEDAI* systemic lupus erythematosus disease activity

### Principal component analysis

To cover as many indices that affect the outcomes of LN with hypothyroidism as possible, factors with *p* < 0.05 were included as input variables for PCA. The Kaiser–Meyer–Olkin value was 0.7 when all the clinical variables were included; meanwhile, the *p*-value of the Bartlett test of primary data was 0.000, indicating that the data were suitable for use in PCA. We removed symptomatic variables and those of which the extract value were too small in the common factor variance table. The model generated nine PCs that explained 74% of the variation within the dataset; two of these, taken together, explained 30% of the variation. From the viewpoint of variance contribution rate, when eigenvalue *λ*_1_ = 3.515, the PC_1_ contribution rate was 15.3%—the highest value—and it contained the most information (When eigenvalue *λ*_2_ = 3.397, the PC_2_ contribution rate was 14.7%). For the nine main PCs (Table 2), the loadings represented the degree of importance of the corresponding compound. For example, the first three degrees of importance of PC_1_ in the sequence were albumin (ALB) > TP > C3; likewise, the first three degrees of importance of PC_2_ in the sequence were SCr > BUN > UA. In focusing on the indices whose loading was obviously higher than those of others, we could clearly see that PC_1_ was mainly about renal functions (including SCr, BUN, and UA); PC_2_ was about serum protein factor (including TP and ALB); PC_3_ was a leukocyte factor; and PC_4_ was a globulin factor. We additionally found that PC_5_–PC_8_ could not be accurately classified as any certain factor bearing a specific meaning, and PC_9_ was an autoantibody factor.

**Table 2:** Component loadings

	PC_1_	PC_2_	PC_3_	PC_4_	PC_5_	PC_6_	PC_7_	PC_8_	PC_9_
WBC	.020	−.023	**.469**	.094	−.006	.035	−.021	−.041	.084
HB	−.067	.206	.080	.018	−.024	−.097	.070	.179	.118
PLT	−.002	.105	.004	−.009	.129	−.237	−.075	.261	.054
N	.032	−.041	**.471**	.064	.009	.023	.007	−.109	.052
L	−.092	−.053	−.009	.017	−.017	.121	−.058	**.405**	.042
TP	.063	**.366**	−.022	.067	−.028	.128	−.017	−.008	−.015
ALB	.064	**.405**	−.058	−.077	−.122	.013	.007	−.048	.001
GLB	.031	.143	.026	.182	.083	.182	−.033	.037	−.023
Scr	**.347**	.092	−.029	−.108	.019	.039	−.009	−.079	−.137
BUN	**.299**	.055	.052	−.142	.033	.021	−.011	−.074	.069
UA	**.311**	.068	.084	.028	.028	.071	.027	.004	−.002
TG	.203	.060	.006	.138	−.109	−.015	.080	.068	−.005
TC	.159	.097	−.114	−.076	−.054	−.012	.030	**.442**	−.152
HDL	−.021	−.023	.057	−.315	.084	.178	.042	.185	−.152
LDL	.080	.135	.021	.000	−.140	−.273	**.354**	−.167	.245
HDLTC	−.147	−.021	.102	−.234	−.009	.225	.014	−.103	−.045
ESR	−.028	−.047	−.010	.128	**.339**	−.115	−.063	.119	−.036
CRP	.051	−.035	.056	−.104	**.442**	−.114	.114	−.242	−.157
C3	.060	.257	−.029	−.107	.063	−.141	−.008	.040	−.174
C4	−.035	−.021	−.011	.108	.060	**−.429**	−.027	−.118	.017
IgG	.057	.107	.012	.104	−.014	**.289**	−.052	−.010	−.019
IgA	−.021	−.099	−.013	−.186	.**461**	.020	.008	.025	.151
IgM	−.109	−.099	.130	**.459**	−.100	.080	.031	−.025	−.195
Anti-dsDNA antibodies	−.023	−.056	−.081	−.121	.138	.016	−.090	.163	.289
Anti-Sm antibodies	.035	.004	−.010	−.059	.004	.028	.**488**	−.039	−.078
Anti-SSA antibodies	.016	−.010	.001	−.060	−.018	.038	−.006	.088	.147
Anti-SSB antibodies	−.010	.013	−.037	−.018	−.051	−.053	.061	−.062	−.057
Anti-U1RNP antibodies	−.023	−.037	.000	−.021	.057	−.008	**.444**	−.027	−.045
Anti-RNP antibodies	−.020	.003	.093	−.057	−.035	−.036	.000	−.051	**.579**

Loading pattern of the thirty clinical values on the nine main principal components. The loadings of the variables most relevant for the component interpretation are bolded

### PCA–logistic regression analysis

**Fig. 1:**
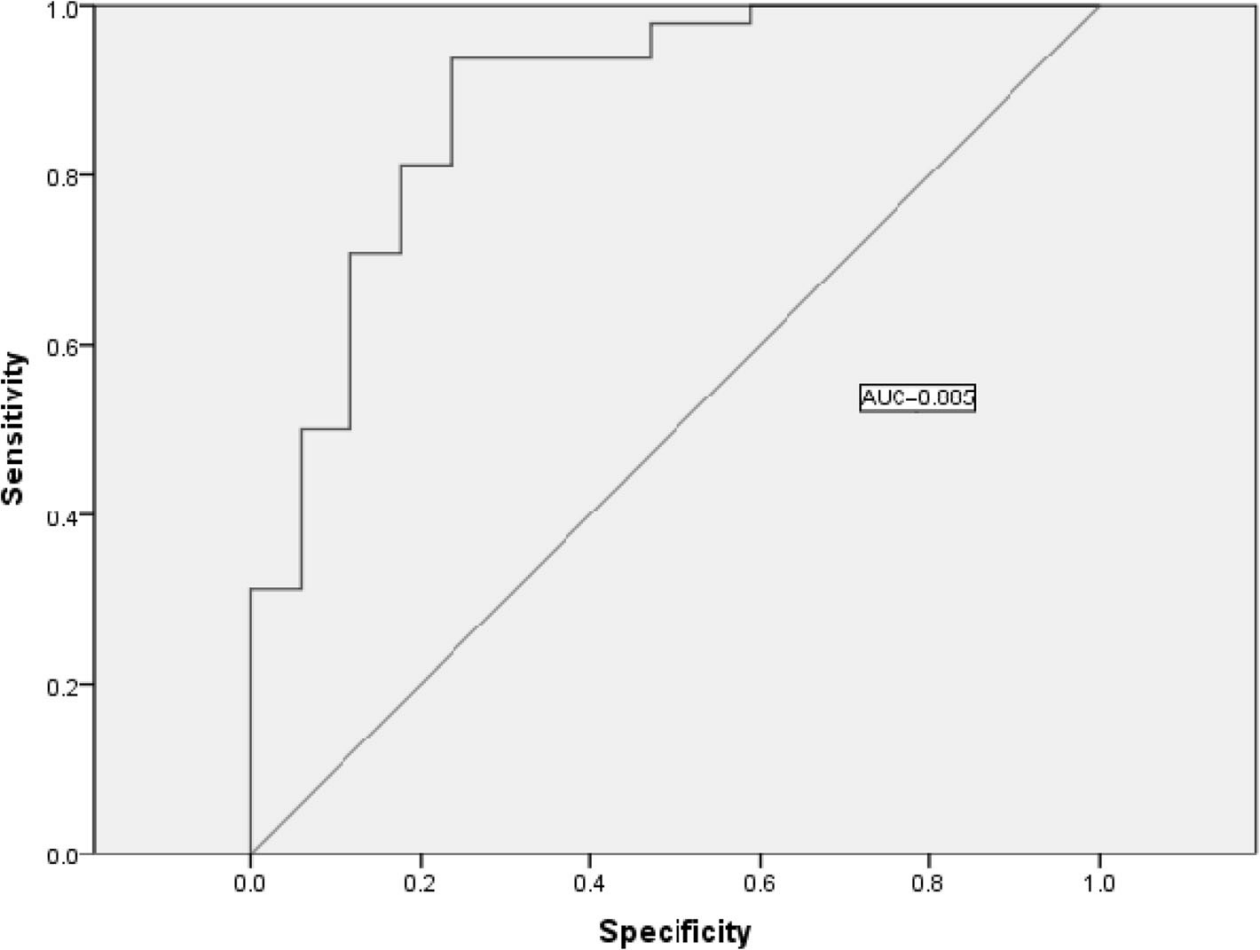
The ROC curve of logistic regression (unadjusted model)

We used the nine PCs as input variables and the clinical outcome (LN with or without hypothyroidism) as a dependent variable in logistic regression modelling. Our analytical results showed that PC_1_, PC_2_, and PC_9_ were the PCs that have a significant influence on whether LN was combined with hypothyroidism (Table 3)—that is to say, SCr, BUN, UA, TP, ALB, and anti-ribonucleoprotein (RNP) antibody might be paramount factors in treating LN with hypothyroidism. It is noteworthy that the Exp(B) of PC_2_ and PC_9_ were 2.361 and 4.724, respectively; these indicate that the correlation between each of these two PCs and LN patients with hypothyroidism was much stronger than that between other pairings. We also generated an ROC (Fig. 1) that was close to the top-left corner of the coordinate system. The area under the ROC curve (AUC) was 0.885 (*p* < 0.001).

**Table 3:** The result of logistic regression analysis

	B	SE	Wals	Sig.	Exp(B)	95% CI of Exp(B)	
						lower limit	upper limit
**PC**_**1**_	−.919	.320	8.249	**.004**	.399	.213	.747
**PC**_**2**_	.859	.322	7.100	**.008**	2.361	1.255	4.442
PC_3_	−.238	.260	.838	.360	.788	.473	1.312
PC_4_	.203	.230	.783	.376	1.225	.781	1.922
PC_5_	−.284	.344	.682	.409	.752	.383	1.478
PC_6_	−.128	.353	.131	.717	.880	.440	1.759
PC_7_	−.443	.293	2.291	.130	.642	.362	1.140
PC_8_	−.111	.411	.073	.787	.895	.400	2.002
**PC**_**9**_	1.553	.593	6.861	**.009**	4.724	1.478	15.096
Constant	0.744	.411	3.272	.070	2.105		

*B* regression coefficient, *SE* standard error, *Wals* wald test, *Sig* significance, *Exp(B)* the exponent of B or relative risk. This is an unadjusted model with all PCs in together. Significant differences are indicated in bold

## Discussion

We applied PCA–logistic regression analysis to demonstrate that three PCs—namely, PC_1_, PC_2_ and PC_9_, which included SCr, BUN, UA, TP, ALB, and anti-RNP antibody—were found to be important clinical variables with respect to LN patients with hypothyroidism. The Exp(B) of PC_2_ and PC_9_ was 2.361 and 4.724, respectively, indicating that the correlation between these two PCs and the outcome was much stronger than that among others.

Previous studies conclude that the most common kidney derangements associated with hypothyroidism are elevated SCr levels, reduced estimated glomerular filtration rate, and water–electrolyte imbalance [14, 15]. Moreover, SCr levels in SLE patients with hypothyroidism were found to be elevated [3]. The current study also showed that renal function indices such as SCr, BUN, and UA are essential factors in whether LN patients are associated with hypothyroidism. Possible mechanisms might include reduced renal perfusion [16], adaptive preglomerular vasoconstriction caused by filtrate overloads [17], and decreased endothelial nitric oxide synthase activity/capacity of the renal vasculature caused by reduced secretion of insulin-like growth factor 1 and vascular endothelial growth factor [18].

Severe hypoalbuminemia was observed in SLE patient with subclinical hypothyroidism [3], correspondingly, we found lower TP and ALB were influential for LN patients with hypothyroidism. Actually, most thyroid hormones are bound to plasma proteins including thyroid-binding globulin (TBG), thyroxine-binding pre-albumin (TBPA) and ALB. While kidney function of LN patients is impaired, TBG, TBPA and ALB are significantly reduced because of severe and persistent proteinuria, thyroid hormone synthesis is also affected by this [19, 20]. Furthermore, the serum hormonal concentration may be altered by changes in the binding capacity of serum proteins, thereby patients with hypoproteinemia may exhibit clinical features and laboratory findings suggestive of hypothyroidism [21, 22].

Additionally, in this study, higher anti-RNP antibody level had massive effect among LN patients with hypothyroidism, which has not been reported before. Anti-RNP antibody reacts with proteins that are associated with U1 RNA and form U1snRNP, autoimmunity to RNP autoantigens is frequently seen in systemic autoimmune diseases including lupus and it may induce the occurrence of renal disease [23–25], thyroid hormone synthesis may be affected by impaired kidney function as mentioned earlier. Moreover, the induction of anti-RNP autoantibodies is associated with the initial clinical manifestations of autoimmune disease, in this case, autoantibodies may lead to thyroid hormone synthesis disorders by damaging the thyroid follicular epithelium [26–29], suggesting that RNP related immune responses may have pathogenic roles in hypothyroidism. Accordingly, those hypotheses deserved to be verified through further mechanism research.

## Conclusions

The principal component analysis (PCA)–logistic regression model approach used herein is a useful statistical method by which to analyse the effects of multiple clinical index interactions in lupus nephritis (LN) patients who also have hypothyroidism. Using this model, we found serum creatinine (SCr), blood urea nitrogen (BUN), blood uric acid (UA), total protein (TP), albumin (ALB), and anti-ribonucleoprotein (RNP) antibody to be particularly vital factors with respect to these patients. What is more, the impact of PC_9_—which mainly involved the anti-RNP antibody—was the strongest among these patients: its Exp(B) was 4.724, the highest among nine principal components. SCr, BUN, UA, TP, ALB, and autoantibody levels are modifiable factors that can be improved through early treatment to improve renal function and strengthen nutrition support, in order to reduce risk among LN patients with hypothyroidism. Ultimately, PCA offers great insights in exploring the influence of clinical variables or measuring the important factors that affect patient outcomes.

## Data Availability

The datasets used and/or analysed during the current study are available from the corresponding author on reasonable request.

## Abbreviations

ALB: Albumin
AUC: Area under the curve
BUN: Blood urea nitrogen
UA: Blood uric acid
Hb: Haemoglobin
LN: Lupus nephritis
PC: Principal component
PCA: Principal component analysis
ROC: Receiver operating characteristic
RNP: Ribonucleoprotein
SCr: Serum creatinine
SLE: Systemic lupus erythematosus
TP: Total protein
TBG: Thyroid-binding globulin
TBPA: Thyroxine-binding pre-albumin
